# Development of PMAxx^TM^-Based qPCR for the Quantification of Viable and Non-viable Load of *Salmonella* From Poultry Environment

**DOI:** 10.3389/fmicb.2020.581201

**Published:** 2020-09-22

**Authors:** Jiawei Zhang, Samiullah Khan, Kapil K. Chousalkar

**Affiliations:** School of Animal and Veterinary Sciences, The University of Adelaide, Roseworthy, SA, Australia

**Keywords:** viability assay, *Salmonella* contamination, poultry production, PMAxx-based qPCR, *Salmonella* load

## Abstract

Determining the viable and non-viable load of foodborne pathogens in animal production can be useful in reducing the number of human outbreaks. In this study, we optimized a PMAxx^TM^-based qPCR for quantifying viable and non-viable load of *Salmonella* from soil collected from free range poultry environment. The optimized nucleic acid extraction method resulted in a significantly higher (*P* < 0.05) yield and quality of DNA from the pure culture and *Salmonella* inoculated soil samples. The optimized primer for the amplification of the *invA* gene fragment showed high target specificity and a minimum detection limit of 10^2^ viable *Salmonella* from soil samples. To test the optimized PMAxx^TM^-based qPCR assay, soil obtained from a free range farm was inoculated with *Salmonella* Enteritidis or *Salmonella* Typhimurium, incubated at 5, 25, and 37°C over 6 weeks. The survivability of *Salmonella* Typhimurium was significantly higher than *Salmonella* Enteritidis. Both the serovars showed moisture level dependent survivability, which was significantly higher at 5°C compared with 25°C and 37°C. The PMAxx^TM^-based qPCR was more sensitive in quantifying the viable load compared to the culture method used in the study. Data obtained in the current study demonstrated that the optimized PMAxx^TM^-based qPCR is a suitable assay for quantification of a viable and non-viable load of *Salmonella* from poultry environment. The developed assay has applicability in poultry diagnostics for determining the load of important *Salmonella* serovars containing *invA*.

## Introduction

Food safety is an important aspect of human health and, therefore; state health departments are routinely tasked to trace back foodborne pathogens for their source of origin. Zoonotic pathogens, such as *Salmonella* and *Campylobacter*, often result in human gastroenteritis after the consumption of contaminated poultry products (Kärenlampi et al., 2007; Ford et al., 2018). Due to consumer demand, there has been an increase in free range poultry production over the years. In a free-range poultry production system, birds have access to the range area outside poultry shed. Chickens are the common host for multiple serovars of *Salmonella enterica*, which naturally colonize their gut. Therefore, infected chickens can shed *Salmonella* to their environment intermittently. In a free range production system, the infected flock can contaminate range soil and result in the infection of the newly introduced flock. Therefore, it is vital to understand the viable and non-viable load of *Salmonella* in the poultry environment in relation to changes in temperature.

Soil type and temperature affect the survivability of *Salmonella* more than moisture level (Underthun et al., 2018). The final distribution of water and bacteria in the soil is influenced by soil texture, pH, temperature, and organic matter (Semenov et al., 2009). Temperature and water activity affect the survivability of *Salmonella* with the thermodynamic activity of water as the main factor in determining the survival. A study has shown that the incidence of *Salmonella* increases during warm seasons (Milazzo et al., 2016). Cross-contamination of the range area may pose a continuous threat to the health of the layer flocks. Therefore, understanding the viability of *Salmonella* in the soil is a crucial factor in devising strategies for its control in poultry production. In Australia, *Salmonella* Enteritidis is considered as an exotic pathogen in commercial poultry. Recently, there have been several *Salmonella* Enteritidis outbreaks on free range layer farms in Australia, which has prompted the mass culling of layer flocks. After mass culling, sheds are decontaminated, but it is often a challenge to decontaminate ranging areas.

Quantitative PCR (qPCR) can be used for quantifying pathogen load in clinical samples; however, primers targeting a specific region of the nucleic acids will also amplify DNA obtained from the non-viable load present in the population. Viability assays, such as live/dead cell viability assay, trypan blue, BacTiter-Glo^TM^ microbial cell viability assay (Promega, Australia), LIVE/DEAD BacLight bacterial viability kit (ThermoFisher Scientific, Australia) and propidium monoazide (PMA) (Banihashemi et al., 2012; Barbau-Piednoir et al., 2014; Jäger et al., 2018) or ethidium bromide monoazide (Nocker et al., 2006; Wang and Mustapha, 2010) based qPCR can be used for quantifying viable microbial load. PMA is a nucleic acid intercalating dye that is cell impermeant and therefore will diffuse only across the cell membrane of dead microbes. After binding to nucleic acids, upon photolysis, the dye covalently reacts with the microbial DNA/RNA thus inhibiting it from the amplification by PCR. Therefore, PMA-based qPCR can be a reliable assay for differentiating viable cells from non-viable population of microbes. However, given the complex nature of the environmental samples, none of them have been optimized for quantifying the viable and non-viable load of *Salmonella* from the environment. In the current study, PMAxx^TM^-based qPCR was optimized for the quantification of *Salmonella* from poultry environment and in the subsequent *Salmonella* survivability experiment, its applicability was tested. Therefore, the three main objectives of the current study were: (A) Optimize method for PMAxx^TM^ based viability qPCR for *Salmonella* quantification from the soil. (B) Understand the effects of temperature on the survivability of *Salmonella* Typhimurium and *Salmonella* Enteritidis in soil. (C) Apply the optimized PMAxx^TM^ based qPCR to quantify the viable and non-viable load of *Salmonella* Typhimurium and *Salmonella* Enteritidis from soil samples incubated at various temperatures.

## Materials and Methods

Pure cultures of *Salmonella* Typhimurium phage type 9 or *Salmonella* Enteritidis (phage-type 7A obtained from Elizabeth McArthur Agriculture Institute, NSW, Australia) were streaked onto nutrient agar (NA; ThermoFisher Scientific, Australia) plates and incubated overnight at 37°C. A single colony was subcultured in Luria Bertani (LB; ThermoFisher Scientific, Australia) broth at 37°C in a shaking incubator until the OD_600_ was approximately 1, which was used for calculating the colony forming unit (CFU) per mL.

### DNA Extraction From Pure Culture of *Salmonella* and *Salmonella* Inoculated Soil

The *Salmonella* DNA extraction from pure culture (*n* = 6) and soil (*n* = 6) was carried out using the QIAamp DNA Mini Kit (Qiagen, Australia) with some modifications in the protocol. Approximately 10^9^ CFU/mL of *Salmonella* broth culture was centrifuged at 19,500 × *g* for 3 min and the pellet was resuspended in 100 μL phosphate buffered saline (PBS) into which 300 μL tissue lysis buffer (ATL) and half spoon of acids washed 106 μm beads (Sigma Aldrich, Australia) were added. The samples were homogenized (Bullet Blender, Next Advance, United States) for 2–3 min. The samples were heated at 70°C for 15 min with occasional mixing and then centrifuged for 1 min at 5,000 × *g* to remove the beads and undissolved materials. The supernatant was transferred to a 1.5 mL tube that contained 50 μL proteinase K (20 mg/mL). After a brief vortexing, 50 μL lysozyme (100 mg/mL) and 300 μL AL lysis buffer were added. The samples were briefly vortexed and incubated at 70°C for 15 min with occasional inversion. To avoid clump formation, the samples were vortexed at each step of adding the reagents. After the incubation, 300 μL ethanol (100%) was added and the lysates were passed through the spin column by centrifuging for 1 min at 6,000 × *g* and the process was repeated for the rest of the lysates. The DNA in the spin columns was washed with 500 μL of each of wash buffers I and II and eluted in 50 μL of buffer ATE as per the manufacturer’s protocol. The extracted DNA was assessed for quality and concentration in a Nanodrop 1000 and stored at −20°C for further use.

The DNA extraction method from soil was optimized based on adding 9 mL of PBS to 1 g of soil. Briefly, 1 g of soil was weighed in 15 mL tubes and inoculated with 10^9^ CFU/mL of *Salmonella* and the samples were mixed thoroughly. The tubes were centrifuged at 900 × *g* for 1 min to settle down the undissolved materials. The maximum supernatants were transferred to 15 mL tubes and centrifuged at full speed (4500 × *g*) for 15 min at 4°C. The bacterial pellet layer was gently resuspended in 100 μL of PBS and transferred to 1.5 mL tubes. The DNA was extracted as described previously. In a separate pilot study, to test the efficiency of the bacterial separation method from soil, the bacterial pellet was resuspended in PBS, serially diluted, plated onto xylose-lysine deoxycholate (XLD; ThermoFisher Scientific, Australia) agar media and incubated overnight at 37°C.

### Optimization of PMAxx^TM^ Step for Distinguishing Between Viable and Non-viable Load

*Salmonella* culture in the LB broth was prepared as described previously. The broth culture samples were prepared as viable and non-viable *Salmonella*. For the non-viable culture preparation, *Salmonella* was exposed to 95°C for 5 min in a dry heat block. To confirm the non-viable *Salmonella*, 100 μL of the culture was plated onto XLD and incubated overnight at 37°C. For determining the optimum concentration of PMAxx^TM^ dye (Biotium, United States), 10 μL of the dye solution (2.5, 5, and 10 mM) was added into 1 mL of the *Salmonella* culture in LB broth (*n* = 6) or left as a control (*n* = 6). The PMAxx^TM^ treated samples were incubated in dark for 10 min on a rocker. After the incubation, the samples were exposed to light (500 volts) at a distance of 20 cm for 15 min with intermittent inversion. The samples were centrifuged at 19500 × *g* for 3 min, the bacterial pellets were suspended in 100 μL PBS and the DNA was extracted as described previously.

### Optimization of Primers and Construction of Standard Curve

Primers were either designed using the NCBI primer software or sourced from literature. Primers were evaluated for their secondary structure characteristics in web based NetPrimer and Beacon Designer software. Short sequence primers (<300 bp) were avoided to reduce the chances of amplifying genomic DNA obtained from non-viable bacterial population in samples treated with PMAxx^TM^. To broaden the scope of this study for detection of a range of *Salmonella enterica* serovars, primer amplifying the fragment of invasion A gene (*invA*; Table 1) was used for the PMAxx^TM^-based qPCR. To determine the specificity and amplification efficiency of the *invA* primer, qPCR was performed on 10-fold serial dilutions (10^8^ to 10^1^) of *Salmonella* DNA extracted from a pure culture (*n* = 3 and repeated twice). To determine the minimum detection limit of the *invA* primer, qPCR was performed on DNA extracted pure culture and from spiked soil samples (*n* = 3 and repeated twice) with different dilutions of *Salmonella* (10^8^ to 10^1^). The qPCR products were assessed by melting curve analysis and then visualized on 2% gel electrophoresis to assess the specificity of the primer pair. For the quantification of *Salmonella* from soil samples, a standard curve was generated from a 10-fold serial dilutions (10^–2^ to 10^–12^) of purified qPCR product (using the QIAquick PCR Purification Kit) of *invA* fragment and used as a reference to determine the load of *Salmonella*. Primer pairs for amplifying *ompA* gene were also evaluated for specificity through qPCR conducted on DNA obtained from pure cultures of *Salmonella* Typhimurium and *Escherichia coli* (Table 1).

**TABLE 1:** Detail of the primers used in optimization of the PMAxx^TM^ -based qPCR assay.

Gene symbol	Primer sequence (5′-3′)	Accession no.	Product size (bp)	Annealing temperature (°C)	Amplification efficiency (%)
*invA*	F: AAACCTAAAACCAGCAAAGG R: TGTACCGTGGCATGTCTGAG	M90846.1	605	59	92
*invA*	F: TGGGGCGGAATATCATGACG R: AGGAAGGTACTGCCAGAGGT	M90846.1	1045	60	80
*ompA*	F: TACGCTGGTGCTAAACTGGGCT R: AGCGCGAGGTTTCACGTTGTCA	NC_003197.2	882	60	83
*ompA*	F: CAGTACCATGACACCGGCTT R: ATTCCAGACGGGTTGCGATT	NC_003197.2	385	60	90

*Specificity of each primer pairs was confirmed through melting curve analysis and gel electrophoresis. Sequence of the invA 605 bp was sourced from literature (Akiba et al., 2011). Among the listed primers in Table 1, invA having 605 bp sequence length had higher amplification efficiency and target specificity. Therefore, primer pair labeled as invA 605 bp was used in the quantification of viable and non-viable load of Salmonella Enteritidis and Salmonella Typhimurium through PMAxx^TM^ -based qPCR from soil samples incubated at 5, 25, and 37°C for up to 6 weeks.*

### Quantitative PCR

The qPCR was performed with the SensiFAST SYBR Hi-ROX Kit (Bioline, Australia) following the manufacturer’s protocol. The master mix was prepared and applied to reaction wells through Corbett CAS1200 robot (Corbett Life Science, Australia). The final reaction volume of 20 μL contained 10 μL SensiFAST SYBR Hi-Rox mix, 1 μL of each of the forward and reverse primers (10 μM concentration), 6 μL RNase-free water and 2 μL of DNA template. The reaction was performed in duplicates in a Rotor-Gene Disc 100 (Qiagen, Australia) with a Rotor-Gene 6000 Thermocycler (Corbett Life Science, Australia). Each run contained no-template control for ruling out external contaminations. The three-step cycling conditions included an initial denaturation at 95°C for 3 min, then 40 cycles of denaturation at 95°C for 5 s and annealing and extension at 59°C (Table 1) and 72°C for 20 and 30 s, respectively. During qPCR cycles, the fluorescence detection was conducted at the end of each annealing step, and a melting curve analysis step (at a ramp from 55 to 95°C) was included to assess the specificity of the amplification.

### Preparation of Soil Samples

Soil from a range area in a free-range production system was collected for assessing the survivability of *Salmonella* Typhimurium and *Salmonella* Enteritidis. To mimic the field conditions, the samples were inoculated with 5 × 10^9^ CFU per 100 g of soil with moisture adjusted to around 15%. The moisture level was adjusted by adding autoclaved water into the soil sample containers and measuring the moisture content with a basic Moisture Analyser Model MJ33 (Mettler Toledo, Switzerland). Each treatment group contained three biological replicates and the experiment was repeated twice to confirm the applicability of the optimized method. The *Salmonella* inoculated soil samples were incubated at 5, 25, and 37°C for 6 weeks and the samples were processed at weekly intervals for quantifying the bacterial load through culture method and PMAxx^TM^-based qPCR.

The moisture content and water activity of the individual soil samples were measured at each sampling time-point. Moisture content is a measure of the total amount of water in a sample. A basic moisture analyzer that consists of both weighing and heating and is used to determine the moisture content of a sample with the loss on drying principle. For measuring the moisture content, approximately 8 g of soil was spread onto a sample pan and measured by a Basic Moisture Analyzer (Model MJ33, Mettler Toledo, Switzerland) following the below formula:

$$\begin{array}{c} \mathrm{Moisture}\mathrm{content}\left(0\ldots -100\%\right)\\ \;=-\frac{\mathrm{wet}\mathrm{weight}-\mathrm{dry}\mathrm{weight}}{\mathrm{wet}\mathrm{weight}}\times 100\% \end{array}$$

The values were recorded as loss in total moisture content.

Water activity (a_w_) is a thermodynamic measurement of the energy of water in a sample. The value indicates how tightly water is bound, structurally or chemically, within a substance. The lower a sample’s water activity, the more tightly bound that water is within the sample. It is measured using a water activity meter (range: 0.00–1.00 a_w_) that assesses the partial vapor pressure of water in a sample and divides it by the standard state partial vapor pressure of water. To measure the water activity, approximately 3 g of soil was dispensed into a sample cup and measured by PawKit Water Activity Meter (Model P06760; AquaLab, Decagon Devices, United States).

### Quantification of *Salmonella* by Culture Method and PMAxx^TM^-Based qPCR

In order to understand the applicability of the optimized PMAxx^TM^-based qPCR in the field conditions, soil samples inoculated with *Salmonella* Enteritidis or *Salmonella* Typhimurium, and incubated at different temperatures (5, 25, and 37°C) were processed at weekly intervals for the quantification of *Salmonella* load through culture method and qPCR. The culture data (obtained by direct plating) were presented as log_10_ CFU/g, while the PMAxx^TM^-based qPCR data were presented as log_10_ *Salmonella* load for viable and non-viable counts per gram of soil samples.

At each sampling time-point, 1 g of the soil samples was mixed into 9 mL of buffered peptone water (BPW; ThermoFisher Scientific, Australia), serially diluted, plated onto XLD agar media and incubated overnight at 37°C. The *Salmonella* count was expressed as log_10_ CFU/g of soil sample. A sub-set of the samples was processed using an enrichment method. For the enrichment method, the BPW samples were incubated overnight at 37°C and a 100 μL was added into 9 mL of Rappaport-Vassiliadis soy peptone broth (RVS; ThermoFisher Scientific, Australia). The RVS samples were incubated overnight at 42°C to allow the selective growth of *Salmonella*. The incubated RVS samples were streaked on XLD plates for the confirmation of *Salmonella* in the soil samples. Positive *Salmonella* samples were scored as 1 and negative as 0 for the determination of the proportion of positive samples.

At each sampling time-point, 1 g of soil from individual samples was weighed into 15 mL tubes containing 9 mL PBS for DNA extraction and PMAxx^TM^-based qPCR. The soil samples were centrifuged at 900 × *g* for 1 min at 4°C to remove the undissolved materials. The supernatants were centrifuged at 4500 × *g* for 15 min at 4°C and the bacterial pellets were resuspended in 1 mL PBS. The samples were treated with PMAxx^TM^ or left as control and processed for DNA extraction and qPCR as described previously. A standard curve was used to quantify the DNA copy number from the cycle quantitation (Cq) values. The qPCR data (log_10_ DNA copy number) were expressed as viable and non-viable load of *Salmonella* per gram of soil samples.

### Statistical Analysis

Data for *Salmonella* CFU/g, the proportion of positive samples and viable and non-viable load/g were analyzed in StatView version 5.0.1.0 by taking *Salmonella* treatment and sampling time-point as main effects. Level of significance was determined at protected least significant difference (PLSD) < 0.05. Mean values ± SD were visualized in GraphPad Prism 8.

## Results

### Optimization of PMAxx^TM^-Based qPCR for the Quantification of Viable and Non-viable Load of *Salmonella*

**FIGURE 1:**
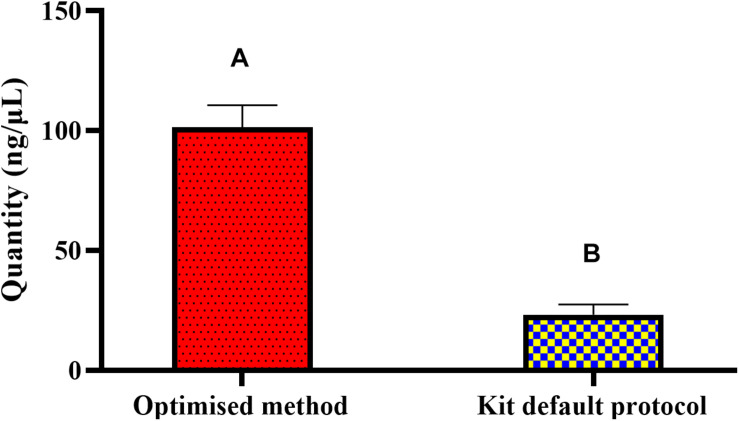
DNA quantity(ng/μL) obtained through the optimized and the standard kit protocol methods from pure culture of *Salmonella*. The optimized extraction method resulted in significantly higher yield of DNA. Different superscripts (^A,B^) across the bars show significant difference.

The optimized method for DNA extraction from pure culture resulted in significantly (*P* < 0.05) higher yield and pure genomic DNA of *Salmonella*. Tested in Nanodrop-1000, 1 × 10^9^ CFU/mL of *Salmonella* broth yielded an average of 101.38 ng/μL DNA with 260/280 and 260/230 ratios of 2.10 and 2.32, respectively (Figure 1). Primer optimization steps showed that primers for the *ompA* gene were non-specific and amplified the genomic DNA of *Escherichia coli* (data not provided). Long size sequence primers (∼1045 bp) designed for the amplification of the *invA* gene were less efficient (80%) compared to the medium size primers (∼605 bp). Therefore, the primers that amplified the 605 bp region of *invA* were used in the subsequent PMAxx^TM^ -based qPCR for quantifying viable and non-viable load of *Salmonella* Enteritidis and *Salmonella* Typhimurium from soil samples incubated at 5, 25, and 37°C for up to 6 weeks. Culture count data obtained from plating the isolated *Salmonella* from the inoculated soil showed no significant effect of the background materials. These data (4 × 10^8^ CFU/mL) compared well with the dose (10^9^ CFU/mL) used for 1 g of soil inoculation.

**FIGURE 2:**
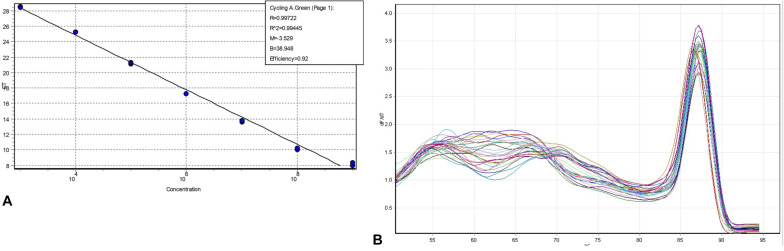
Amplification efficiency and melting curve analyses of *invA* determined in 10-fold serial dilutions of DNA. **(A)** PCR performed on *Salmonella* DNA extracted from a pure culture with 1 × 10^9^ CFU/mL. **(B)** Melting curve of *invA* (605 bp) in DNA obtained from 10-fold serially diluted (10^8^ to 10^1^ CFU/g) *Salmonella* spiked soil samples.

The qPCR results showed that the amplification efficiency of the *invA* primers in the 10-fold serial dilutions of DNA extracted from *Salmonella* was 92% with *R*^2^ value as 0.99 (Figure 2A). The melting curve analysis of the amplicon showed a single peak, thereby confirming the specificity of the *invA* primers in the DNA obtained from *Salmonella* spiked soil samples (Figure 2B). The gel results confirmed that the minimum detection limit of *invA* in pure culture and soil samples spiked with 10-fold serial dilutions of viable *Salmonella* subsequently treated with PMAxx^TM^ was 10^1^ and 10^2^ *Salmonella* cells, respectively (data not shown).

**FIGURE 3:**
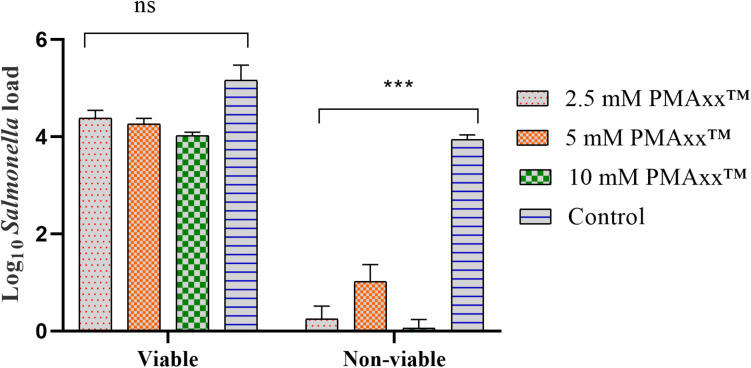
Effects of different concentrations (2.5, 5, and 10 mM) of PMAxx^TM^ on the amplification of target DNA obtained from the viable and non-viable culture of *Salmonella*. The data showed that PMAxx^TM^ efficiently inhibited the amplification of the target DNA obtained only from the non-viable culture of *Salmonella*. This trial was repeated three times and an average of the data was presented. For obtaining non-viable cells, *Salmonella* in LB broth was heated at 95°C for 5 min, and a 100 μL plated on XLD agar and incubated overnight at 37°C confirmed that the heat treatment was effective in killing 100% of *Salmonella* in the broth. Asterisks (^∗∗∗^) denote significant difference at *P* < 0.0001, while ns denotes non-significant difference.

To assess the inhibitory effects of PMAxx^TM^ on the target DNA amplification, qPCR was performed on DNA obtained from viable and non-viable *Salmonella* culture treated with 3 different concentrations of PMAxx^TM^. The qPCR data (expressed as log_10_ *Salmonella* load) showed that the PMAxx^TM^ concentration did not significantly (*P* > 0.05) inhibit the amplification of DNA obtained from the viable *Salmonella* (Figure 3). However, PMAxx^TM^ concentrations significantly (*P* < 0.05) inhibited the amplification of DNA obtained from the non-viable culture of *Salmonella* (Figure 3). These data showed that 5 μL of 2.5 mM PMAxx^TM^ per 500 μL of *Salmonella* culture was sufficient for use in PMAxx^TM^-based qPCR assay to discriminate between the viable and non-viable *Salmonella* load. The significantly higher *Salmonella* load in the control samples of the non-viable group confirmed that PMAxx^TM^ penetrated across the cell membrane of compromised cells only.

### Survivability of *Salmonella* Assessed by Culture Method

**FIGURE 4:**
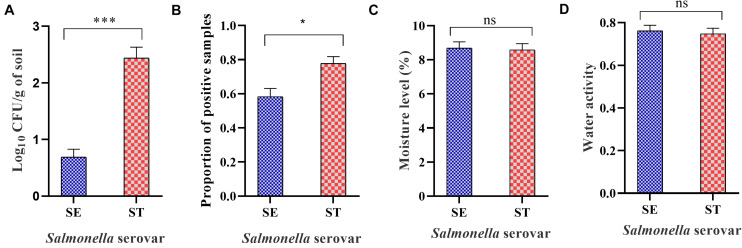
Overall load of *Salmonella* Enteritidis (SE) and *Salmonella* Typhimurium (ST) in soil incubated at different temperatures and sampled at weekly intervals. The samples were processed through direct plating for *Salmonella* load determination. Samples that turned negative through the direct plating were enriched for the qualitative assessment of *Salmonella*. **(A)** Mean log_10_ CFU/g of SE and ST in soil over 6-week of incubation time period. **(B)** Mean proportion of *Salmonella* positive samples of SE and ST in soil incubated at different temperatures. **(C)** Mean moisture level of soil inoculated with SE or ST **(D)** Mean water activity (a_w_) of soil inoculated with SE or ST. Asterisks (^∗∗∗^) and (^∗^) denote significant difference at *P* < 0.0001 and *P* < 0.01, respectively, while ns denotes non-significant difference.

The culture based method showed that the overall survivability of *Salmonella* was significantly higher for *Salmonella* Typhimurium compared with *Salmonella* Enteritidis (Figure 4A) despite the non-significant difference in moisture level and water activity between the two treatment groups (Figures 4C,D). Samples that were negative by direct plating and processed by the enrichment method showed higher survivability for *Salmonella* Typhimurium strain compared with *Salmonella* Enteritidis strain in soil samples incubated at 25 and 37°C (Figure 4B).

**FIGURE 5:**
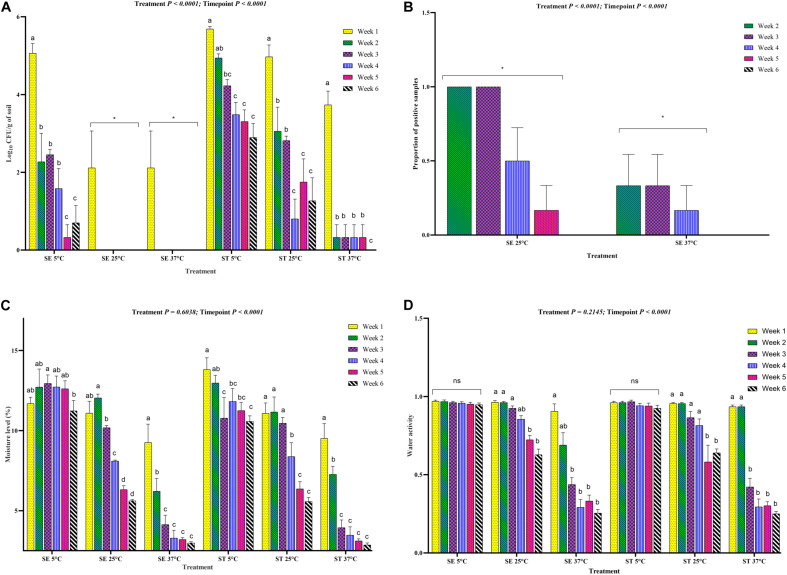
Load of *Salmonella* Enteritidis (SE) and *Salmonella* Typhimurium (ST) in soil incubated at three different temperatures for 6 weeks. **(A)** Log_10_ CFU/g of soil of SE and ST affected by storage temperatures (5, 25, and 37°C). **(B)** Proportion of positive soil samples for SE and ST determined through the enrichment method. Only, the samples that did not show any *Salmonella* growth after direct plating were enriched. **(C)** Moisture level of the *Salmonella* inoculated soil samples incubated at 5, 25, and 37°C. **(D)** Water activity of the *Salmonella* inoculated soil samples incubated at 5, 25, and 37°C. The *Salmonella* inoculated soil samples were processed for *Salmonella* load through culture, *Salmonella* detection through enrichment method and moisture and water activity determination at weekly intervals for up to 6 weeks of incubation time period. Asterisk (^∗^) denotes significant difference at *P* < 0.01, while ns denotes non-significant difference. Different superscripts (^a,b,c,d^) show significant difference within each treatment group at each sampling timepoint.

The survivability of *Salmonella* Enteritidis and *Salmonella* Typhimurium (log_10_ CFU) in soil was significantly affected by storage temperature, moisture level and water activity (Figures 5A–D). *Salmonella* Typhimurium survived at a significantly higher level than *Salmonella* Enteritidis at 5, 25, and 37°C (Figure 5A). Throughout the experiment, the survivability of both the serovars decreased significantly, whereas *Salmonella* Enteritidis could not be quantified by culture method from soil samples incubated at the 25 and 37°C from week 2 onward (Figure 5A). Furthermore, both the *Salmonella* Enteritidis and *Salmonella* Typhimurium in soil samples incubated at 5°C survived better than the samples incubated at 25 and 37°C. Therefore, the data confirmed that the *Salmonella* Typhimurium strain had a better capability to resist to temperature and moisture driven changes compared to the *Salmonella* Enteritidis strain.

To determine if there was relatively low viable *Salmonella* in the samples with no growth after direct plating, samples were further tested by the enrichment method. The results showed that *Salmonella* Enteritidis was not detectable in the week 5, and 6 soil samples incubated at 37°C, while *Salmonella* Typhimurium was detected until week 5 of incubation (Figure 5B). At 25°C, *Salmonella* Typhimurium was quantified from the soil samples until week 6 of incubation (Figure 5A). Therefore, the data confirmed that the culturability was driven by water activity and moisture level in the soil with a higher loss in moisture at 25 and 37°C (Figures 5C,D).

**FIGURE 6:**
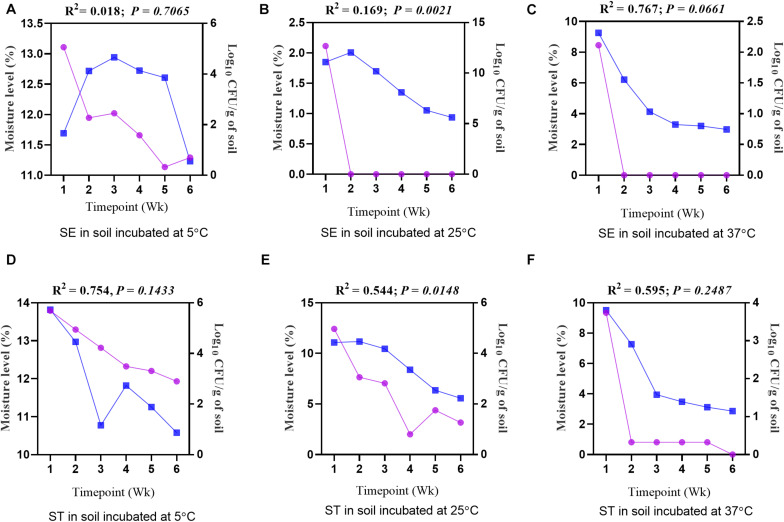
Regression analysis showing a positive correlation between the moisture level (%) and the load of *Salmonella* Enteritidis (SE) and *Salmonella* Typhimurium (ST) in soil incubated at 5, 25, and 37°C. **(A)** Soil inoculated with SE and incubated at 5°C. **(B)** Soil inoculated with SE and incubated at 25°C. **(C)** Soil inoculated with SE and incubated at 37°C. **(D)** Soil inoculated with ST and incubated at 5°C. **(E)** Soil inoculated with ST and incubated at 25°C. **(F)** Soil inoculated with ST and incubated at 37°C. Blue lines show moisture level (%), while purple lines show log_10_ CFU/g of soil.

To investigate how moisture level (%) and water activity influenced the survivability of different *Salmonella* serovars, regression analysis between the moisture level and log_10_ CFU/g of soil samples was performed. Overall, for both the *Salmonella* Enteritidis and *Salmonella* Typhimurium treatment groups at all temperatures, the log_10_ CFU/g decreased with a decline in the moisture level (Figures 6A–F) and water activity (Table 2). For the samples incubated at 25 and 37°C, the decline of log_10_ CFU/g was highly associated with the decrease in the moisture level, while lower consistency of results was recorded at 5°C. Especially for *Salmonella* Enteritidis samples incubated at 5°C, there was a weak correlation between the log_10_ CFU/g and the moisture level.

**TABLE 2:** Correlation of water activity and *Salmonella* load (log_10_ CFU/g of soil).

Treatment	*R*^2^	*P*-value
*Salmonella* Enteritidis in soil incubated at 4°C	0.474	0.0001
*Salmonella* Enteritidis in soil incubated at 25°C	0.181	0.0020
*Salmonella* Enteritidis in soil incubated at 37°C	0.633	0.0069
*Salmonella* Typhimurium in soil incubated at 4°C	0.707	0.0001
*Salmonella* Typhimurium in soil incubated at 25°C	0.461	0.0036
*Salmonella* Typhimurium in soil incubated at 37°C	0.333	0.0397

*At each sampling time-point, water activity of the individual samples was measured by PawKit Water Activity Meter as per the manufacturer’s instructions.*

### Survivability of *Salmonella* Assessed by PMAxx^TM^ -Based qPCR

**FIGURE 7:**
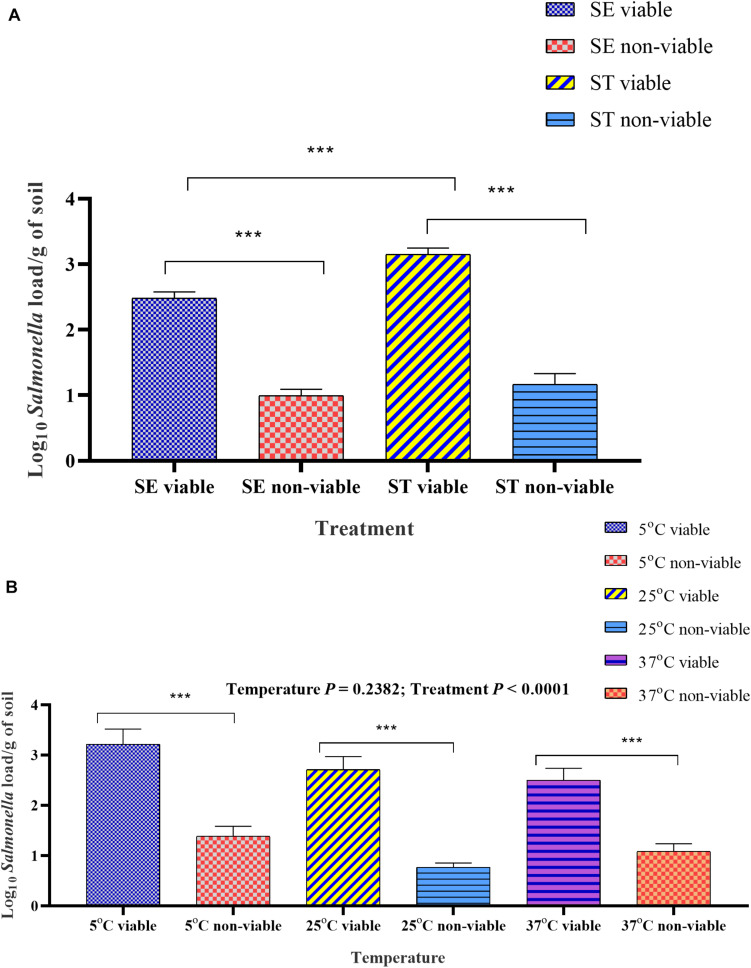
Overall viable and non-viable load quantified by PMAxx^TM^ -based qPCR from *Salmonella* inoculated soil affected by temperature. **(A)** Mean log_10_ viable and non-viable load of *Salmonella* Enteritidis (SE) and *Salmonella* Typhimurium (ST) in soil samples incubated at 5, 25, and 37°C and processed at weekly intervals over 6-week of incubation time period. **(B)** Mean log_10_ viable and non-viable load of SE and ST affected by temperature. Samples were treated either with PMAxx^TM^ or left as control before the extraction of DNA. The DNA copy number was calculated through the standard curve and the data were presented as log_10_ *Salmonella* load. Asterisks (^∗∗∗^) denote significant difference at *P* < 0.0001.

The optimized PMAxx^TM^ -based qPCR was used to assess the survivability of *Salmonella* serovars in soil samples incubated at three different temperatures. The overall viable and non-viable load in the *Salmonella* Enteritidis inoculated soil was significantly lower than *Salmonella* Typhimurium (Figure 7A). The viable load was significantly higher in soil samples incubated at 5°C compared to the samples incubated at 25 and 37°C (Figure 7B) due to moisture level driven changes.

**FIGURE 8:**
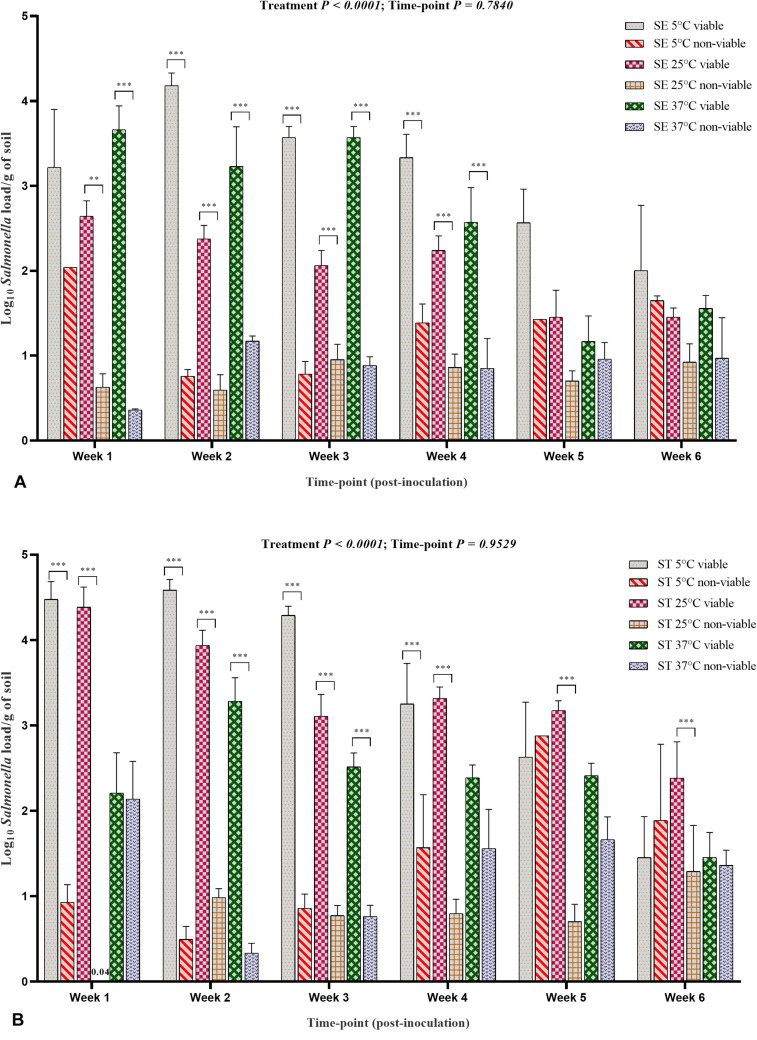
Viable and non-viable load of *Salmonella* Enteritidis (SE) and *Salmonella* Typhimurium (ST) in soil samples. **(A)** SE load. **(B)** ST load. The samples were incubated at 5, 25, and 37°C and processed at weekly intervals for PMAxx^TM^ -based qPCR. Asterisks (^∗∗∗^) show significant difference (*P* < 0.0001) between the viable and non-viable load of *Salmonella* affected by each incubation temperature at each sampling timepoint.

The PMAxx^TM^ -based qPCR data for both the *Salmonella* Enteritidis and *Salmonella* Typhimurium treatment groups showed that the survivability of *Salmonella* decreased throughout incubation period (Figures 8A,B). The data at weekly intervals and incubation specific temperature showed some discrepancy in terms of higher load at week 2 compared with week 1 possibly due to the unequal distribution of *Salmonella* in the soil samples. The viable load of *Salmonella* Enteritidis and *Salmonella* Typhimurium in the samples incubated at 5°C was significantly higher than the samples incubated at 25 and 37°C (Figures 8A,B) due to moisture level driven changes. The viable load decreased with the increase in storage temperature and sample incubation period.

**FIGURE 9:**
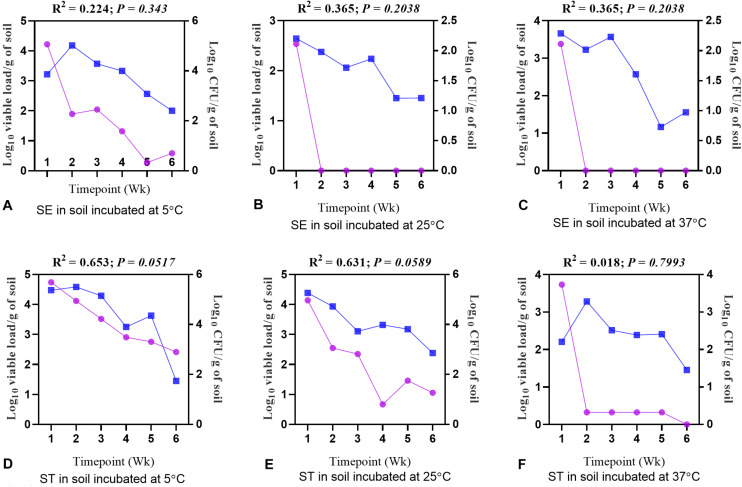
Correlation between log_10_ CFU/g and log_10_ viable load of *Salmonella*. **(A)** Soil inoculated with *Salmonella* Enteritidis (SE) and incubated at 5°C. **(B)** Soil inoculated with SE and incubated at 25°C. **(C)** Soil inoculated with SE and incubated at 37°C. **(D)** Soil inoculated with *Salmonella* Typhimurium (ST) and incubated at 5°C. **(E)** Soil inoculated with ST and incubated at 25°C. **(F)** Soil inoculated with ST and incubated at 37°C. Blue lines represent log_10_ viable load, while purple lines represent log_10_ CFU per gram of soil.

The CFU and viable load data obtained from different treatment groups were compared to understand a correlation between the culture method and the optimized PMAxx^TM^ -based qPCR. There was a significant positive correlation between the CFU and viable load data (Figures 9A–F). The decline in CFU was positively correlated with the qPCR data. The PMAxx^TM^ -based qPCR was more sensitive in quantifying the viable load of *Salmonella* serovars as compared to the culture method used in the study. For both the *Salmonella* Enteritidis and *Salmonella* Typhimurium samples incubated at 5°C, the correlation between CFU and the viable load was significantly stronger compared with the samples incubated at 25 and 37°C due to higher moisture content.

## Discussion

The main objective of this study was to optimize a PMAxx^TM^ -based qPCR for quantification of viable and non-viable *Salmonella* from environmental samples and to test the optimized assay on the artificially contaminated samples. The findings showed that the optimized qPCR can be accurately used for differentiating viable and non-viable *Salmonella* in soil samples collected from the free-range egg farm environment.

The QIAamp Fast DNA Stool Mini Kit (Qiagen, Australia) was used for the extraction of *Salmonella* DNA from pure cultures and *Salmonella* inoculated soil samples. As the manufacturer’s protocol did not yield high quality and quantity of DNA and was not applicable for the inclusion of the PMA step hence, the optimized method for DNA extraction was used in this study. The optimized method in the current study was based on microbe separation from the samples through gradient centrifugation. The qPCR results showed no effects of background materials in soil samples on amplification efficiency. This showed that the optimized method developed in this study removed the background materials during bacterial pelleting from soil samples. The *invA* primers used during this study showed that the primer pair was highly specific for target amplification with high efficiency (92%). The data also showed that its minimum detection limit was 10^2^ *Salmonella* per gram of soil. Primer optimization for the amplification of *ompA* gene showed that these primers were non-specific by amplifying DNA obtained from *Escherichia coli*.

PMA has been used in distinguishing viable from non-viable cells of bacteria in pure culture (Nocker et al., 2006; Li and Chen, 2013) and from food/water matrices (Dinu and Bach, 2013; Kibbee and Örmeci, 2017; Li et al., 2017; Jäger et al., 2018). Similarly, PMA has also been used to differentiate live viruses from the dead population (Bellehumeur et al., 2015). However, the method has not been optimized for complex materials such as soil. Also, previous literature presented data as Cq values rather than viable and non-viable load (Li and Chen, 2013; Li et al., 2015). Therefore, the method optimized during this study is accurate and rapid that can be used to test the presence of viable *Salmonella* from complex soil matrices.

In the current study, the data showed that 10 μL of 2.5 mM PMAxx^TM^ into 1 mL of the *Salmonella* culture was sufficient to differentiate viable cells from the non-viable cells. The DNA extracted from the non-viable bacteria was minimally amplified by qPCR. Studies have reported different concentrations of PMA. For example, 10 μL of 2.5 mM PMA in 0.5 mL of culture samples was used (Liang et al., 2011). However, in the current study, there was no significant difference in the load of *Salmonella* obtained from the viable culture treated with 2.5, 5, and 10 mM PMAxx^TM^. The concentration used in the current study is the recommended concentration from the manufacturer of the improved version of PMA, which is called PMAxx^TM^. If used in the high throughput diagnostic assay platforms, the lower concentration of PMAxx^TM^ can save significant costs required for veterinary diagnostics. Data in the current study showed that the PMAxx^TM^ concentration had no significant inhibitory effects on the amplification of DNA obtained from viable *Salmonella*.

In the subsequent applicability steps of the PMAxx^TM^ -based qPCR, the CFU and qPCR data showed that the survivability of *Salmonella* Typhimurium and *Salmonella* Enteritidis was driven by incubation temperature, moisture level and water activity. The data showed that *Salmonella* Typhimurium had significantly higher capability to survive in soil compared with the *Salmonella* Enteritidis in the conditions applied during this study. However, it is worth to note that survivability of different strains of the same serovars of *Salmonella* may vary in different conditions. Significantly higher survivability at 5°C showed that both the *Salmonella* Enteritidis and *Salmonella* Typhimurium can remain viable for longer periods at 5°C in soil. The significant role of moisture and water activity in the survivability of *Salmonella* shows that the survival of *Salmonella* in the soil in dry and hot summer might be lower due to hot temperatures and low moisture levels. The decrease in the moisture level has been proven as a factor responsible for decline in viable count (Marsh et al., 1998). Nevertheless, our data showed that compared to the *Salmonella* Typhimurium, the *Salmonella* Enteritidis viable load declined at a significantly higher rate when the moisture level and water activity declined. The findings were consistent when the trial was repeated. A higher proportion of positive samples for *Salmonella* Typhimurium compared with *Salmonella* Enteritidis from the enriched samples further proved that the viability of *Salmonella* Enteritidis strain was more susceptible to changes in temperature, water activity and moisture level. A positive correlation between the CFU and PMAxx^TM^ -based qPCR data obtained in the current study is in agreement with the findings of Shah et al. (2019) who quantified the viable count of *Salmonella* Newport through a culture method and PMA-qPCR from heat treated poultry amended soil.

A sharp decline in the survivability of both the serovars after 1 week of incubation at 37°C was due to a significantly higher loss of moisture and water activity at this temperature. In the current study, keeping the moisture level around 15% was to simulate real field conditions where the temperature in summer in South Australia can reach up to 45°C with moisture level quite low. A higher moisture content favors the survivability of bacteria (Cools et al., 2001), where *Salmonella* Newport can survive in soil for longer periods (Shah et al., 2019). The *Salmonella* Enteritidis strain used in this study was isolated from a commercial egg farm. Recently, several *Salmonella* Enteritidis infected commercial free range egg flocks were culled in Australia because *Salmonella* Enteritidis infection in poultry is categorized as exotic and notifiable. Our previous epidemiological investigation suggested that *Salmonella* Typhimurium was persistent in the range area of free range farms during winter (Gole et al., 2017). After *Salmonella* Enteritidis infection of the flocks, the poultry sheds are typically cleaned but, it is challenging to clean the ranging area of a free range poultry farm. The persistence of *Salmonella* Enteritidis or *Salmonella* Typhimurium in range area can result in reinfection of current flock or infection of the newly introduced flock. The assay developed in this study can be used for the rapid detection of *Salmonella* Enteritidis and *Salmonella* Typhimurium from soil samples and can provide useful information to the industry in making decisions on restocking flocks after mass culling. The data on the survival of *Salmonella* Typhimurium and *Salmonella* Enteritidis in soil samples are useful for mitigating the risks on farms and also for developing on farm biosecurity measures to avoid future on farm *Salmonella* outbreaks. The reduction of *Salmonella* in food animals and in a farm environment is essential to reduce human infection. This study was conducted on soil samples collected from a small number of egg farms located in the South Australia region, therefore; further studies are required to investigate the effects of different soil types on the survival of *Salmonella*. Soil type and temperature can influence the persistence of *Salmonella* (Garcia et al., 2010; Underthun et al., 2018). For the persistence of *Salmonella* in soil, humidity has been identified as an important meteorological factor (Hwang et al., 2020).

*Salmonella* can form biofilm when exposed to harsh environmental conditions (De Oliveira et al., 2014) such as soil. Biofilm is an assemblage of microorganism associated with the cell surface, which on the one hand can be the attachment to the surface of the materials in the poultry production system or on the other hand, the ability of the biofilm has been confirmed as one of the reasons that the foodborne pathogen can exist in the system continuously (Wang et al., 2013). Our study revealed that when the soil was inoculated with *Salmonella* and incubated at 5°C, *Salmonella* survived better compared with the samples incubated at higher temperature due to moisture level and water activity driven changes. This finding is in agreement with the previous report (Garcia et al., 2010). The possible reason might be that the biofilm was formed as a barrier to protect bacteria in a harsh environment (De Oliveira et al., 2014), however; further studies are necessary to confirm this hypothesis. *Salmonella* can be exposed to extreme temperature fluctuations including freezing and thawing. Freezing and thawing cycle can significantly affect bacterial cell viability and induce viable but non-culturable (VBNC) state with compromised ability to form a biofilm (Rocard et al., 2018). In this experiment, *Salmonella* Enteritidis and *Salmonella* Typhimurium remained undetected by culture method from week 5 onward, but the qPCR assay was able to detect the viable bacteria from the soil at week 6. This suggests that the low moisture level and high temperature can also induce the VBNC state of *Salmonella*.

The PMAxx^TM^ -based qPCR data showed that the PMA penetrated through the compromised cell membrane of *Salmonella* thereby inhibiting the DNA amplification through qPCR. The positive correlation graphs between the CFU and viable count data at 5, 25, and 37°C for *Salmonella* Enteritidis and *Salmonella* Typhimurium showed that the optimized assay correlated well with the culture method. The viable counts obtained through the PMAxx^TM^ -based qPCR from the soil sampled at weekly intervals showed that the optimized qPCR was sensitive in detecting the load of *Salmonella* compared to the culture method.

In conclusion, the PMAxx^TM^ -based qPCR optimized in this study was sensitive in determining a load of *Salmonella* in soil samples. Both the culture based method and the optimized qPCR data showed that the *Salmonella* Typhimurium survived better in soil than *Salmonella* Enteritidis. Future studies would be able to use this optimized assay in conditions, such as the efficacy of sanitizers against *Salmonella* in poultry production. In poultry and food industries, the quantification of viable *Salmonella* from feces, dust, egg, meat, food products, or other complex mixture of inorganic materials could be tested through our optimized method.

## Data Availability Statement

The raw data supporting the conclusions of this article will be made available by the authors, without undue reservation.

## Author Contributions

All authors listed have made a substantial, direct and intellectual contribution to the work, and approved it for publication.

## Conflict of Interest

The authors declare that the research was conducted in the absence of any commercial or financial relationships that could be construed as a potential conflict of interest.
